# Hyperbaric Oxygen Induces Late Neuroplasticity in Post Stroke Patients - Randomized, Prospective Trial

**DOI:** 10.1371/journal.pone.0053716

**Published:** 2013-01-15

**Authors:** Shai Efrati, Gregori Fishlev, Yair Bechor, Olga Volkov, Jacob Bergan, Kostantin Kliakhandler, Izhak Kamiager, Nachum Gal, Mony Friedman, Eshel Ben-Jacob, Haim Golan

**Affiliations:** 1 The Institute of Hyperbaric Medicine, Assaf Harofeh Medical Center, Zerifin, Israel; 2 Research and Development Unit, Assaf Harofeh Medical Center, Zerifin, Israel; 3 Sackler School of Medicine, Tel-Aviv University, Tel-Aviv, Israel; 4 Nuclear Medicine Institute, Assaf Harofeh Medical Center, Zerifin, Israel; 5 School of Physics and Astronomy, The Raymond and Beverly Sackler Faculty of Exact Sciences, Tel-Aviv University, Tel-Aviv, Israel; 6 Neurology Department, Assaf Harofeh Medical Center, Zerifin, Israel; 7 Center for Theoretical Biological Physics, Rice University, Houston, Texas, United States of America; University of Münster, Germany

## Abstract

**Background:**

Recovery after stroke correlates with non-active (stunned) brain regions, which may persist for years. The current study aimed to evaluate whether increasing the level of dissolved oxygen by Hyperbaric Oxygen Therapy (HBOT) could activate neuroplasticity in patients with chronic neurologic deficiencies due to stroke.

**Methods and Findings:**

A prospective, randomized, controlled trial including 74 patients (15 were excluded). All participants suffered a stroke 6–36 months prior to inclusion and had at least one motor dysfunction. After inclusion, patients were randomly assigned to "treated" or "cross" groups. Brain activity was assessed by SPECT imaging; neurologic functions were evaluated by NIHSS, ADL, and life quality. Patients in the treated group were evaluated twice: at baseline and after 40 HBOT sessions. Patients in the cross group were evaluated three times: at baseline, after a 2-month control period of no treatment, and after subsequent 2-months of 40 HBOT sessions. HBOT protocol: Two months of 40 sessions (5 days/week), 90 minutes each, 100% oxygen at 2 ATA. We found that the neurological functions and life quality of all patients in both groups were significantly improved following the HBOT sessions while no improvement was found during the control period of the patients in the cross group. Results of SPECT imaging were well correlated with clinical improvement. Elevated brain activity was detected mostly in regions of live cells (as confirmed by CT) with low activity (based on SPECT) – regions of noticeable discrepancy between anatomy and physiology.

**Conclusions:**

The results indicate that HBOT can lead to significant neurological improvements in post stroke patients even at chronic late stages. The observed clinical improvements imply that neuroplasticity can still be activated long after damage onset in regions where there is a brain SPECT/CT (anatomy/physiology) mismatch.

**Trial Registration:**

ClinicalTrials.gov NCT00715897

## Introduction

Intensive functional therapy and rehabilitation programs for post stroke patients are considered essential for maximizing the patients' quality of life [1], [2]. Unfortunately, these programs are often just partially successful, and additional therapeutic approaches towards metabolic recovery of affected cerebral tissues are called for. While a considerable amount of preclinical research supports the use of hyperbaric oxygen therapy (HBOT) for post-stroke damaged brain tissue, so far, only 5 articles reported controlled clinical trials of HBOT for stroke patients. These studies, in which the treatment started during the early-acute phase immediately after stroke, yielded non conclusive and somewhat contradicting results [3], [4], [5], [6], [7]. In contrast, a recent phase-I study evaluating the effect of HBOT on chronic neurological deficiencies (due to traumatic brain injury) revealed promising results [8]. However, to date the effects of HBOT on neurological deficiencies due to stroke during the late-chronic phase (the focus of the current report) have not yet been investigated in a prospective randomized trial.

Years of clinical experience revealed that the dramatic spontaneous recovery from stroke occurs mainly within the first 30 days, though moderate and severe stroke survivors continue to improve for at least 90 days [9]. Most of the recovery involves brain regions rendered dysfunctional, but not dead [10]. Accumulated data from visualizations of these non-active (stunned) regions indicates that they may persist alive but dysfunctional for months, even years, after the acute injury [11], [12], [13]. It was proposed that the oxygen supply to these under-active neurons was low due to stroke damage to blood vessels in these regions, leading to oxygen deficiency, anaerobic metabolism and ATP depletion [14], [15]. The decreased oxygen level not only causes reduction in the neuronal activity but also prevents angiogenesis to replace the stroke-damaged blood vessels and the generation of new synaptic connections. Since 1 cm^3^ of normal brain tissue contains about 1 km of blood vessels, high oxygen supply is essential for repair of the stunned regions. Indeed, as has been demonstrated by previous studies, an increase in dissolved oxygen has several beneficial effects in damaged brain tissues [13], [16], [17], [18], [19], [20]. Transport of oxygen to glial mitochondria, the main sites of oxygen utilization, follows oxygen release from erythrocytes into the plasma and then diffusion of the blood-dissolved oxygen across the Blood-Brain Barrier (BBB). Breathing oxygen under hyperbaric conditions has been shown to be a potent means of increasing arterial oxygen tension and consequently the brain oxygen tension [20], [21], [22]. For example, at 2ATA (atmospheres absolute), the plasma O_2_ partial pressure rises above 1,110 mmHg. Hence, it is reasonable to expect that HBOT can be an efficient (and clinically feasible) method for increasing tissue/cellular oxygenation and thus effectively evoking neuroplasticity in the chronically non-active areas during the late post-stroke phase.

Many physiological pathways, each with a different characteristic time, are spontaneously activated following the onset of stroke. Therefore, a challenging question to be addressed considers the optimal time lapse after stroke to start the HBOT procedure. It should also be kept in mind that signals and chemical cues associated with cell death during the acute stage of stroke might, in fact, promote repair during recovery [23] and can be negatively affected by premature application of HBOT. Unlike the case of preclinical animal studies, in clinical practice it is not feasible to apply the HBOT immediately at the stroke onset. Thus, HBOT procedure can practically begin either at the degenerative or at the regenerative stage. One can assume that any added energy during the degenerative stage could further increase the unwanted, post-injury damage. On the other hand, elevated oxygen supply during the regenerative stage would supply the energy needs for the innate brain repair processes. The differences in the time lapse between stroke onset and HBOT application in previous studies are likely to be the reason for the contradictive results obtained for HBOT application during the acute phase after stroke [3], [4], [5], [6], [7]. The aim of the current study was to evaluate the effects of HBOT started at the late-chronic phase after the acute stroke.

## Methods

The study was performed as a prospective, randomized, controlled, two-group trial. The population included patients of ages 18 years or older, who had either ischemic or hemorrhagic stroke 6–36 months prior to their inclusion. All patients had to have at least one motor dysfunction. Exclusions were based on chest pathology incompatible with HBOT, inner ear disease, claustrophobia and inability to sign informed consent. Additional exclusions were based on dynamic neurologic improvements during the last month (based either on objective measurements by external evaluator or on subjective statement by the patients). Smoking was not allowed during the study. All patients signed written informed consent; the protocol was approved by the local Helsinki committee. The study was conducted in the hyperbaric and research units of Assaf-Harofeh Medical Center, Israel.

### Protocol and End Points

**Figure 1:**
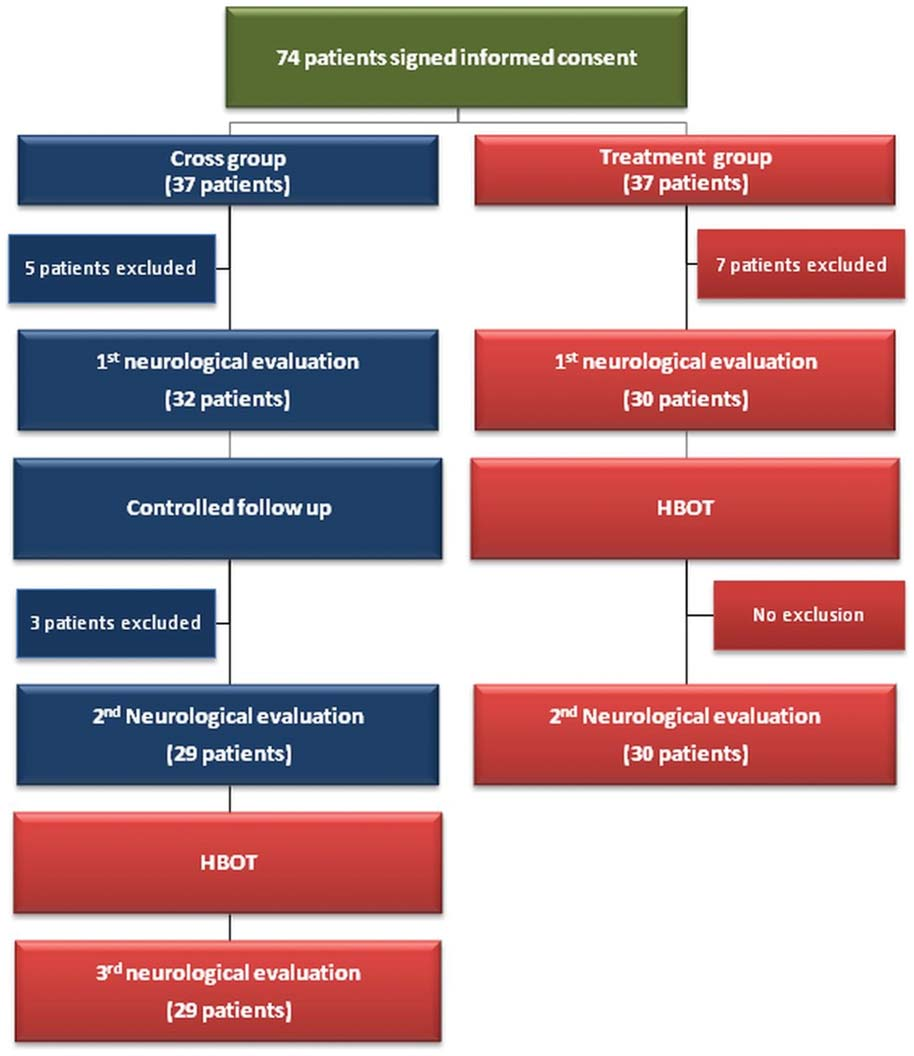
Flowchart of the patients in the study.

After signing an informed consent form, the patients were invited for baseline evaluations. Included patients were randomized into two groups (1∶1 randomization): a treated group and a cross group. The neurologic functions as evaluated by National Institutes of Health Stroke Scale (NIHSS) [24], [25], ability to perform activities of daily living (ADL) [26], and brain metabolism as visualized SPECT were the primary endpoints of the study. The secondary end point of the study included Quality of life evaluation. The patients in the treated group were evaluated twice – at baseline and after 2 months of HBOT treatment. Patients in the cross group were evaluated three times: baseline, after 2 months control period of no treatment, and after consequent 2 months of HBOT sessions (Figure 1). The post-HBOT neurological evaluations as well as the SPECT scans were performed more than 1 week (1–3 weeks) after the end of the HBOT protocol. The following HBOT protocol was practiced: 40 daily sessions, 5 days/week, 90 minutes each, 100% oxygen at 2ATA. The detailed clinical study protocol (Protocol S1), randomization and placebo consideration (Text S1), copy of the informed consent (Form S1), as well as CONSORT 2010 checklist of information (Checklist S1) are attached as supporting information.

### Neurologic Evaluation

The clinical severity of the stroke was blindly assessed by a trained physician according to the NIHSS [24], [25]. The ADL were evaluated by a questionnaire covering the following functions: bathing, dressing, grooming, oral care, toileting, walking, climbing stairs, eating, shopping, cooking, managing medications, using phone, housework, doing laundry, driving and managing finances [26]. For each criterion, the patient defined whether he/she is independent, needs help, dependent or does not do at all (range: 0(best)-51(worst)).

### Brain Functional Imaging and Analysis

Brain single photon emission computed tomography (SPECT) was conducted with 925–1,110 MBq (25–30 mCi) of technetium-99methyl-cysteinate-dimmer(Tc-99m-ECD)at 40–60 min post injection using a dual detector gamma camera (ECAM or Symbia T, Siemens Medical Systems) equipped with high resolution collimators. Data was acquired in 3-degree steps and reconstructed iteratively with Chang method (*μ* = 0.12/cm) attenuation correction [27]. Visual analysis was conducted by fusing pre- and post-treatment studies that were normalized to pre-treatment whole brain activity. SPECT images were reoriented into Talairach space using NeuroGam (Segami Corporation) for identification (based on visual inspection) of abnormal perfusion regions and in order to compute volume rendered brain images.

More specifically, the assessment was done independently by two nuclear medicine physicians who compared the scans and graded them as either: 1 = no change, 2 = mild change and 3 = significant change. This was done “blindly” (without pre-conditioned information about the patients). “No change” was assigned to no visual difference in the number or size of perfusion deficits; “mild change” to a reduction in number or size of perfusion defects; “significant change” to a global perfusion increment in addition to diminution of defect numbers or size. Differences in evaluation were resolved after mutual reviewing. A comparison of the SPECT results with anatomical imaging CT was conducted in order to evaluate the extent of perfusion deficit in relation to the anatomical lesion. All SPECT analysis were done while blinded to the laboratory and clinical data.

### Quality of Life Evaluation

Quality of life was evaluated by the EQ-5D questionnaire [28]. EQ-5D essentially consists of 2 pages: the EQ-5D descriptive system and the EQ visual analogue scale (EQ-VAS). The EQ-5D descriptive system covers mobility, self-care, usual activities, pain/discomfort and anxiety/depression. The EQ-VAS records the respondent’s self-rated health on a vertical, visual analogue scale (range: 0(worst)-100(best)).

### Statistical Analysis

The statistical analysis considerations are detailed in Appendix A. SAS software (version 9.1; SAS Inc.) was used. Continuous data is expressed as means ± STD (standard deviation) and compared by unpaired t-test for inter-group comparison and by paired t-test for intra-group comparison. Categorical data is expressed in numbers and percentages and compared by chi-square test. P values<0.05 were considered statistically significant. All randomly allocated patients were included in the safety analysis and those who had post-baseline assessment were included in efficacy analyses.

### Scatter Plot Analysis of the Clinical Scores

The analysis aims to better quantify and compare changes in the clinical scores, while taking into consideration the high patient-to-patient variability. The idea was to inspect, for each patient at each time stage, the scaled relative differences in each of the clinical scores. More specifically, we calculated for a specific patient (j) the scaled relative difference SRD_j_, defined as:
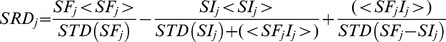



Where SF_j_ is the value of a clinical score at the end of the time stage (either treatment or control), and SI_j_ is the score at the beginning of the time stage. We note that the symbol<> indicates average over the values of the patients in the group. For example, <SF_j_> means the average of SF_j_ over all patients (j) that belong to the group. The abbreviation STD means the standard deviation between the values of the patients in the group. This analysis enables quantitative inspection of the changes in the clinical scores as is further explained and illustrated in Text S2. We note that the results can be further signified when the averaged difference (<SF_j_–SI_j_>) is not divided by STD(SF_j_–SI_j_).

## Results

The study included 74 patients (August 2008-October 2010). 7 patients from the treated group and 8 patients from the cross group were excluded: 8 refused the SPECT, 3 had no measurable paresis, 1 had a medical problem, 1 had a stroke during the control period, and 2 refused to quit smoking (Figure 1).

Twenty four patients (80%) from the treated group had a history of ischemic stroke; of those, 17(71%), 3(13%), 2(8%) and 2(8%) patients were classified as TOAST 1, 2, 3 and 4, respectively. Twenty five patients (86%) from the cross group had ischemic stroke; of those, 18(72%), 3(12%), 2(8%) and 2(8%) patients were classified as TOAST 1, 2, 3 and 4, respectively; p = 0.8 for comparison of the TOAST classification between the groups. Of the 6 patients (20%) in the treated group that had hemorrhagic stroke, 5(83%) had anterior circulation stroke; and of the 4 patients with hemorrhagic stroke in the cross group, 3(75%) had anterior stroke. Baseline patients’ characteristics are summarized in Table 1.

**Table 1:** Baseline patients' characteristics.

	Treated Group(n = 30)	Cross Group(n = 29)	P Value
Age (years)	61±12	63±6.3	0.28
Males/Females	22/8	17/12	0.23
Years of education	14.2±3.7	15.1±3.3	0.39
Time from Stroke (years)	1.49±0.83	1.48±0.79	0.94
Ischemic stroke	24 (80%)	25 (86%)	0.8
Hemorrhagic stroke	6 (20%)	4 (14%)	0.75
Diabetes	10 (33.3%)	12 (41.4%)	0.52
Hypertension	24 (80%)	22 (75.9%)	0.7
Ischemic heart disease	6 (20%)	6 (20.7%)	0.94
Hyperlipidemia	24 (80%)	24 (82.8%)	0.8
History of convulsions	5 (16.7%)	2 (6.9%)	0.09
History of smoking	10 (33.3%)	9 (31%)	0.7
**Medications**			
Aspirin	11 (36.7%)	14 (48.3%)	0.24
Clopidrogel	10 (33.3%)	7 (24.1%)	0.43
Warfarin	3 (10%)	5 (17.2%)	0.91
Statins	23 (76.7%)	23 (79.3%)	0.8
Anti-convulsive	6 (20%)	3 (10.3%)	0.14
Anti-Hypertensive	21 (70%)	22 (75.8%)	0.43
Glucose lowering drugs	9 (30%)	10 (34.5%)	0.7
Anti-Depressants	7 (23.3%)	9 (31%)	0.13

* Data presented as Mean ± standard deviation.

### Neurologic Evaluation

**Figure 2:**
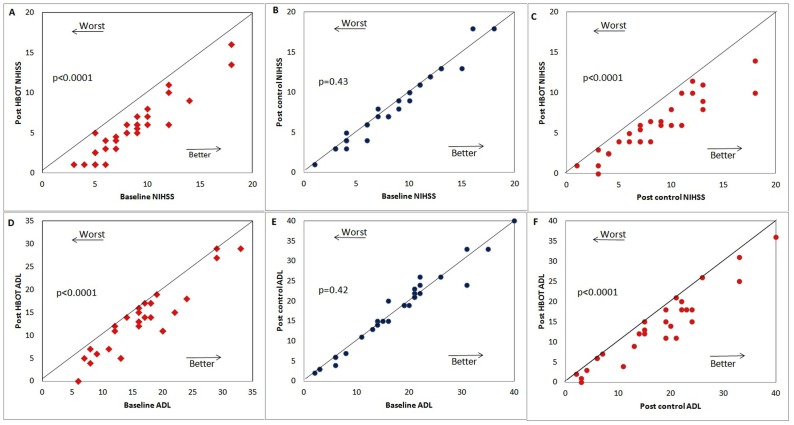
The results of the neurological evaluation. Each point represents a patient. (**A–C**) NIHSS score: (**A**) Scores of the treated group patients before and after the HBOT period (B) Scores of the cross group before and after the control (no treatment) period. (C) Scores of the cross group after the HBOT period. (**D–F**) The same as (A)-(C) for the activities of daily living (ADL) scores. We note that the lines indicate the diagonal. ***Abbreviation***: NIHSS =  National Institutes of Health Stroke Scale; ADL =  Activities of Daily Living.

The results of the neurological evaluations, including the NIHSS and the ADL and the quality of life estimates EQ-5D and EQ-VAS, are summarized in Table 2. Details of the parameter estimates, significance levels and confidence intervals for NIHSS and ADL are presented in Figure 2.

**Table 2:** Summary of the results of the National Institutes of Health Stroke Scale (NIHSS), activities of daily living (ADL) and quality of life questionnaire (EQ-5D and EQ-VAS).

	Treatment group				Cross group				
	Baseline	Post HBOT	P_1_	P_2_	Baseline	Control period	Post HBOT	P_1_	P_3_
**NIHSS**	8.53±3.62	5.52±3.59	<0.0001	0.004	8.71±4.11	8.34±4.25	5.85±3.44	0.43	<0.0001
**ADL**	16.1±6.52	12.77±7.26	<0.0001	0.02	17.38±9.49	17.45±9.53	13.82±8.75	0.42	<0.0001
**EQ- 5D**	9.3±1.36	7.67±1.33	<0.0001	0.009	8.78±1.55	8.64±1.69	7.57±1.51	0.122	<0.0001
**EQ- VAS**	4.93±1.62	6.45±1.50	<0.0001	0.016	5.14±2.25	5.34±2.27	6.79±1.85	0.053	<0.0001

* Data presented as Mean ± standard deviation.

***Abbreviations***: NIHSS =  National Institutes of Health Stroke Scale; ADL =  activities of daily living; EQ =  Evaluation of Quality of life evaluation by the EQ-5D descriptive system and the EQ visual analogue scale (EQ-VAS). HBOT =  Hyperbaric Oxygen Therapy.

P_1_ =  p value compared to baseline in the same group. P_2_ = p value compared to the cross group after the control period. P_3_ = p valus compared to the 2^nd^ evaluation at the end of the control period.

#### NIHSS

Clinical evaluations revealed statistically significant improvements in the NIHSS measures following treatment both in the HBOT-treated group (Figure 2_a_; p = 0.004 compared to control) and in the HBOT-treated cross group (Figure 2_c_; p<0.0001 compared to pre-HBOT). The significance of these improvements is further noticeable when compared to the control (non-treatment) period of the cross group during which the scores did not change at all (Figure 2_b_): p = 0.43 compared to baseline.

#### ADL

Clinical evaluations revealed statistically significant improvements in the ADL score following treatment both in the HBOT-treated group (Figure 2_d_; p<0.001 compared to control) and the cross group after the cross to the HBOT-treated (Figure 2_f_; p<0.0001 compared to pre-HBOT). The significance of these improvements is further noticeable when compared to the control (non-treatment) period of the cross group during which there was no change in the ADL scores (Figure 2_e_; p = 0.42 compared to baseline).

### Scatter Plot Analysis of the Neurological Evaluations

**Figure 3:**
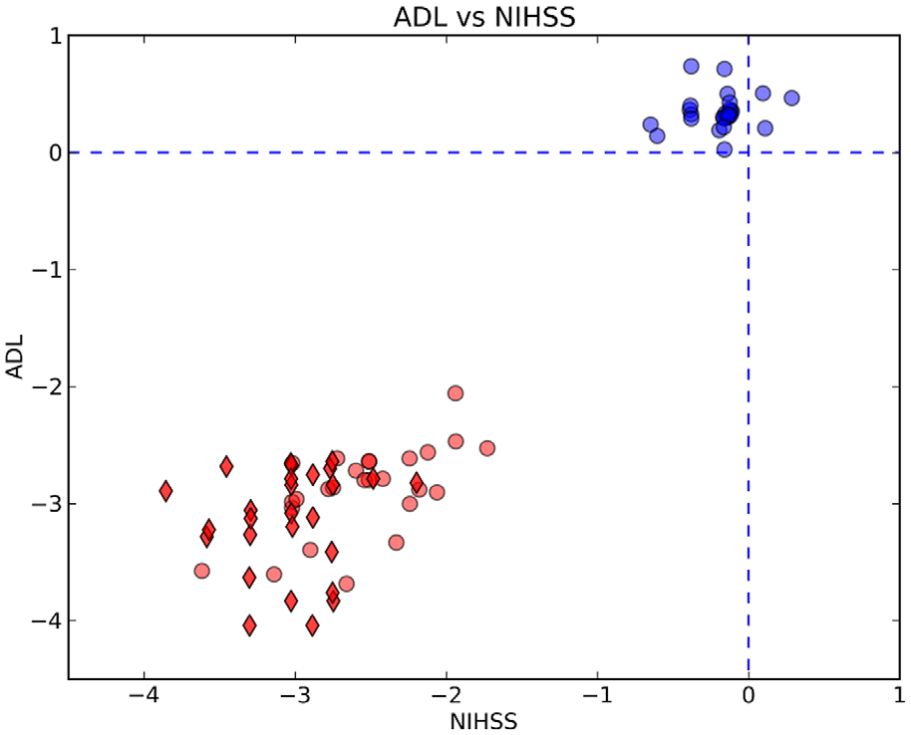
Scatter plot analysis of the changes in the combined neurological evaluations. The scatter plot shows changes in the NIHSS and ADL scores in terms of the scaled relative differences as is defined in the methods section (averaged difference (<SF_j_–SI_j_>) is not divided by STD(SF_j_−SI_j_)). The color code is – changes during the treatment periods for the HBOT treated group (red diamonds), changes during for the HBOT-treated cross group (red circles) and changes during the control (non-treatment) period of the HBOT-treated cross group (blue circles).

The statistical significance of the improvements following the treatment periods is noticeable in the scatter plot analysis represented in Figure 3 and further detailed in Appendix B. In particular, the results show that the combined score of all patients improved following HBOT, while remaining unchanged during the control period.

### Quality of Life

The effect on the quality of life is summarized in Table 2. The EQ-5D score significantly improved following treatment, both for the HBOT-treated group (p<0.0001 compared to baseline) and the HBOT-treated cross group (p<0.0001 compared to pre-HBOT), while there was no improvement following the control period (p = 0.122 compared to baseline, p = 0.009 for comparison between the groups). Similar results were obtained for the EQ-VAS evaluations as is summarized in Table 2. More specifically, the EQ-VAS score significantly improved following treatment, both for the HBOT-treated group (p<0.0001 compared to baseline) and the HBOT-treated cross group (p<0.0001 compared to pre-HBOT), while there was no significant improvement following the control period (p = 0.053 compared to baseline, p = 0.016 for comparison between the groups).

### Brain Functional Imaging- rCBF SPECT Imaging

All brain SPECT evaluations were completed for 29 patients in the treated group and for 28 in the cross group. Comparison of brain activity improvement following the HBOT revealed that 55% of the treated group had significant improvement after HBOT and 35% had mild improvement. In the cross group, during the first (control) period 36% had mild improvement and only 6.2% had significant improvement (p<0.001). After HBOT, the cross group demonstrated 43% significant improvement and 29% mild improvement (p<0.001) (data not shown in tables).

The improvements in the SPECT were mostly in regions showing noticeable discrepancy between the CT and SPECT–the earlier mentioned stunned regions of low activity living cells. The following examples of three typical patients illustrate the associations between the improvements in the patients’ clinical conditions and evaluations and the changes in their brain activity (indication of the activation of neuroplasticity) as reflected by changes in their corresponding SPECT images:

#### Example-1

**Figure 4:**
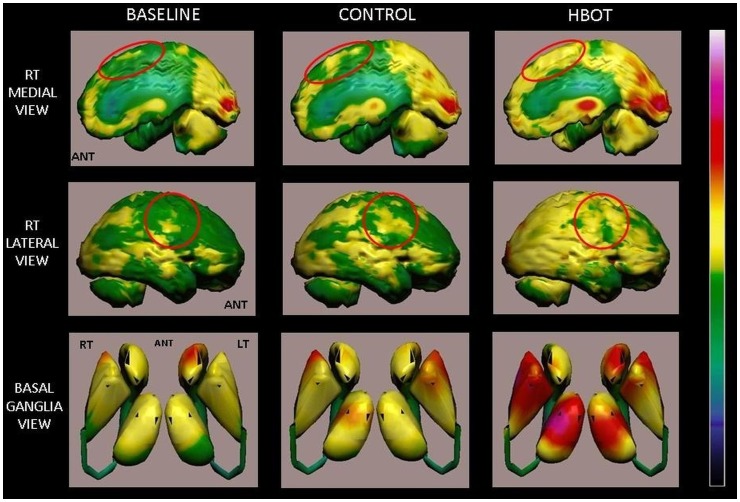
Volume rendered Brain SPECT perfusion maps of Example 1. The results are of a patient in the cross group, suffering from left hemiparesis due to ischemic stroke that occurred 1 year prior to inclusion in the study. Baseline and control volume rendered brain perfusion views show diffuse hypoperfusion in the right hemisphere involving the fronto-parietal region and right postero-medial frontal (right motor cortex), right medial parietal and posterior-parietal (sensory cortex and associative motor cortex) (red circles). The HBOT SPECT scan done at the end of HBOT treatments shows disappearance of the perfusion deficits that were still demonstrated at the end of the control period. In addition, a significant global cortical and subcortical (basal ganglia and thalamic nuclei) perfusion improvement is seen.

**Figure 5:**
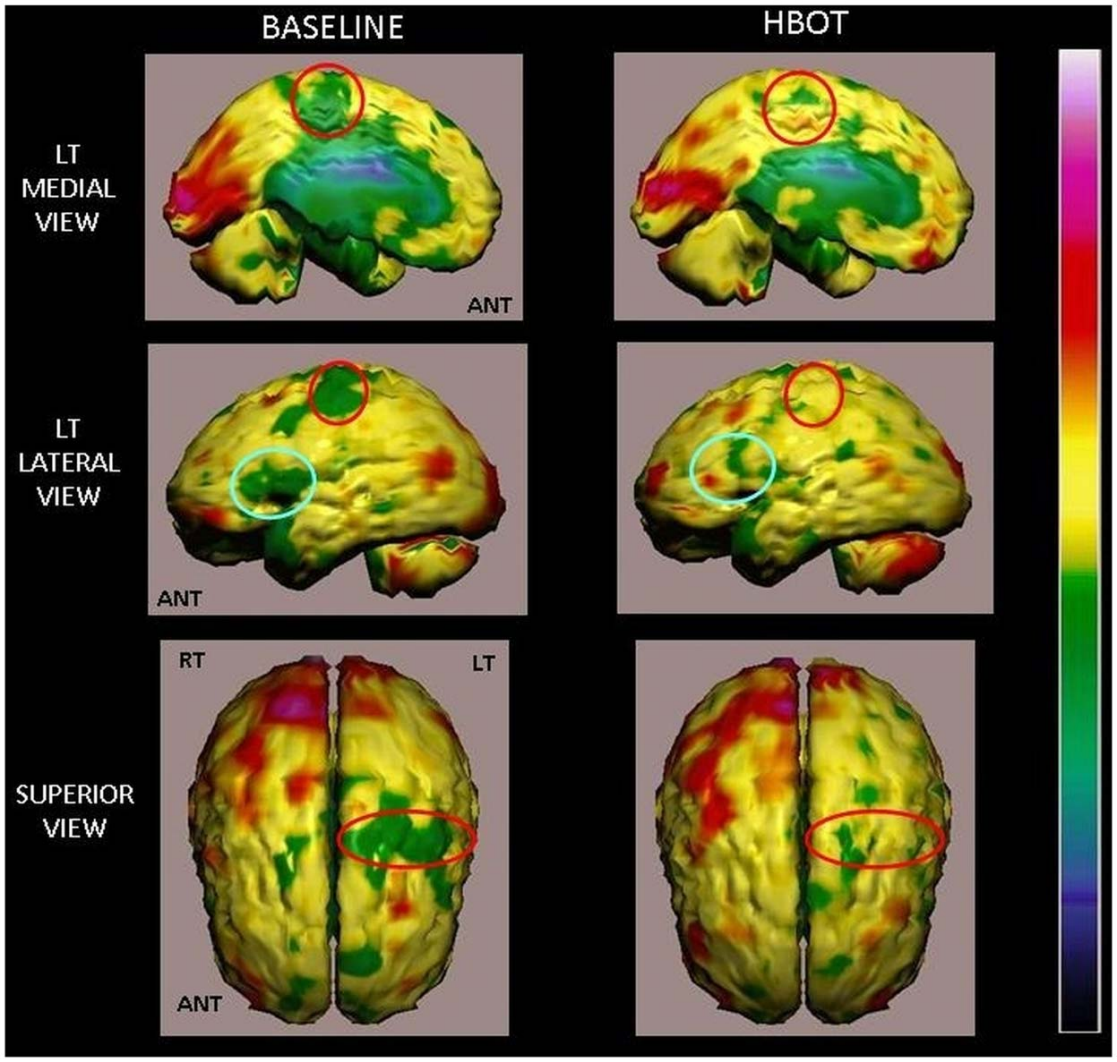
Volume rendered Brain SPECT perfusion maps of Example 2. The results are of a patient in the treated group, suffering from right hemiparesis due to ischemic stroke that occurred 14 months prior to her inclusion in the study. Comparison of pre- and post-hyperbaric treatment SPECT scans. These SPECT images demonstrate significant improvement of perfusion deficits in the left hemisphere involving the medial and posterolateral frontal area (motor cortex, red circles) and lateral inferior frontal region (Broca's area, blue circles) in comparison to the baseline SPECT. HBOT SPECT findings correlate positively with the patient's improved motor and verbal functions.

Baseline brain SPECT images demonstrating hypoperfusion in the right fronto-parietal region, right postero-medial frontal and posterior-parietal perfusion lesions with no significant changes after the control period (Figure 4). In comparison, the SPECT after HBOT demonstrated disappearance of the perfusion lesions. Global cortical and subcortical perfusion improvement was seen (Figure 5).

These SPECT images are of a 61y old woman from the cross group, suffering from left hemiparesis due to ischemic stroke that occurred 1 year prior to inclusion. Baseline NIHSS showed minor facial paresis, no ability to hold her left hand against gravity, some ability to hold her left leg against gravity for less than 5 seconds and mild-to-moderate sensory loss. In ADL, she needed help in bathing, dressing and climbing stairs. She was unable to do any housework. After HBOT, she was able to hold her hand and leg against gravity without significant sensory loss. She could move her fingers, was independent in bathing, dressing, shopping and cooking.

#### Example-2

SPECT images at the end of HBOT demonstrating significant improvement of perfusion deficit in the left hemisphere (Figure 5) involving the medial and posterolateral frontal area (motor cortex) and lateral inferior frontal area (Broca's area). These images are from a 62y old woman from the treated group suffering from right hemiparesis due to ischemic stroke that occurred 14 months prior to inclusion. Baseline NIHSS showed no movement in her right arm, some effort against gravity in her right leg, mild-moderate aphasia, alexia and mild-moderate dysarthria. In ADL, she was completely dependent in bathing and dressing and needed help in transferring, walking, climbing stairs and eating. After HBOT, she could move her right hand against gravity, move fingers, and hold her leg against gravity. She regained speech (almost fluent) and reading capabilities. In ADL, she was able to walk, climb stairs and eat by herself. She was not dependent in bathing and dressing.

#### Example-3

**Figure 6:**
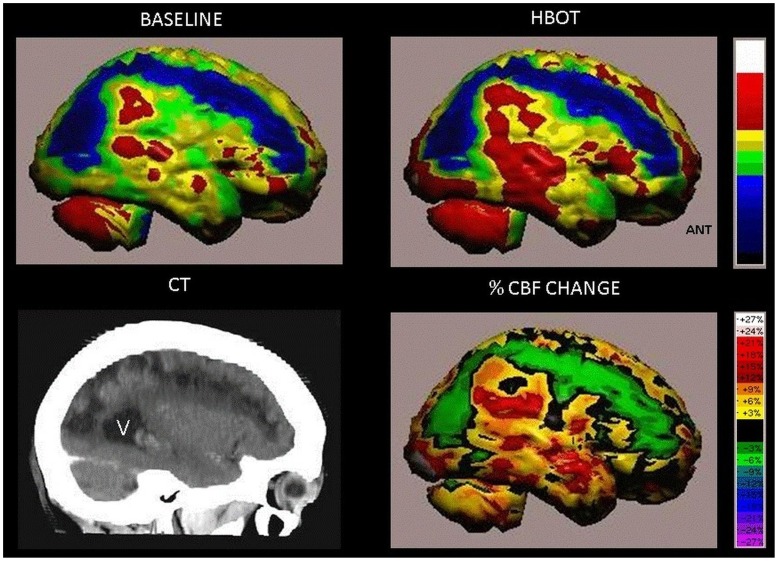
Volume rendered Brain SPECT perfusion maps of Example 3. The results are of a patient in the treated group suffering from left hemiparesis due to ischemic stroke that occurred 26 months prior to inclusion in the study. The brain perfusion maps (upper two images) show the infracted brain (deep blue color) involving the right antero-postero-lateral frontal, right superior-parietal and right parieto-occipital regions. Curved sagittal view in CT MIP reconstruction of the brain shows the anatomical stroke area (left lower image, V = posterior horn of right ventricle). The peri-infarct region show improved perfusion as demonstrated by HBOT image (right upper image). Quantitation of the cerebral blood flow (CBF) change (delta between baseline and HBOT) is demonstrated in the right lower image.

SPECT images demonstrating improvement in the peri-infarct region following HBOT. The images are of a 64y old woman from the treated group suffering from right hemiparesis due to ischemic stroke that occurred 26 months prior to inclusion. After treatment, the leg hemiparesis was resolved, her hand function improved significantly but she did not regain all fine motor skills. Figure 6 shows improvement in the peri-infarct region.

### Safety

Six patients had mild-moderate barotrauma of the middle ear. After several days of rest they returned and completed the protocol. Two patients with a history of epileptic seizures prior to their inclusion in the study had mild episodes of convulsion (consciousness was fully maintained) during the study. Both patients were already treated with anti-epileptic drugs prior to their inclusion.

## Discussion

In the current study, the effect of HBOT on chronic neurological deficiency due to stroke was evaluated in a prospective, randomized controlled study. Statistically significant improvements were obtained following treatment for almost all treated patients from both the HBOT-treated group and the HBOT-treated cross group (with no false negative), as was evaluated by NIHSS, ADL, brain SPECT and life quality. The significance of the improvements in this chronically debilitated population of patients is further noticeable when compared to the lack of improvement during the control (no-treatment) period of the cross group (with no false positive).

This is the first prospective, randomized clinical study evaluating the effect of HBOT in the late post-stroke period (6 months to 3 years after the acute event). There are two major reasons for selecting this study population. First, by carefully selecting patients with chronic stable neurological deficiency we were able to avoid unexpected changes in their condition. In this regard, the selection proved very useful since the control group demonstrated neurological stability with no outliers. The second reason was, as discussed in the introduction, to test our hypothesis that the optimal time for the HBOT procedure should be during the regenerative and not during the degenerative stage. While it is not possible to mark a clear line between the regenerative and the degenerative phases [23], it is quite clear that 6 months after the acute event in a stable patient the degenerative process has ended. As mentioned in the introduction, the differences in initiation times and protocols of HBOT may explain contradictive results in previous studies, where HBOT was used in the early phase after stroke [3], [4], [5], [6], [7]. The recent publication by Harch et al., evaluating the effect of HBOT on chronic neurological deficiencies due to traumatic brain injury, also supports the use of HBOT in the late stage after the acute insult [8].

The issue of “how to handle the control group” was discussed by a multidisciplinary team including physicians specializing in hyperbaric medicine, physicists specializing in neuronal-glia interactions and the ethics committee. The patients can tell if pressure is increased or not, so the pressure must be increased also in the control group. The only way to administer “placebo” of HBOT is to bring the patients to the hyperbaric chamber and to increase the environmental pressure to an extent that the patients will feel it in their ears. The minimal pressure needed to gain such a feeling should be 1.3 ATM. Henry’s law states: “the amount of a given gas dissolved in a given type and volume of liquid is directly proportional to the pressure of that gas in equilibrium with that liquid”. Thus, hyperbaric environment significantly increases the dissolved oxygen pressure even if a person holding his breath [29]. Compressed air at 1.3 ATA increases the plasma oxygen tension by at least 50% and that is certainly notable. There are many case reports illustrating significant effects following small increase in air pressure [30], [31], [32]. Moreover, even a slight increase in partial pressure, such as, for example, to 1.05 ATM at altitude 402 m below sea level (the Dead Sea), can lead to noticeable physiological effects [33], [34], [35], [36], [37]. However, it should be kept in mind that oxygen is not a drug, and because it is metabolized mainly in the mitochondria, there is no simple dose-response curve.

Since increasing the pressure even without adding oxygen can also increases the dissolved oxygen partial pressure, the only way to maintain normal (placebo) levels of dissolved oxygen is to supply air with lower than normal level of oxygen, which we deemed unethical. To partially compensate for this inherent limitation, the patients in the cross group started with a two-month control period of no treatment, at the end of which they were crossed to two months of HBOT sessions. To gain better validity of the results, we used the scatter plot analysis of the changes in the combined neurological evaluations. The scatter plots (figure 3) show changes in the NIHSS and ADL scores in terms of the scaled relative differences, as is defined in the methods section. In that analysis, summarized in figures 2 & 3, the correlation between changes NIHSS & ADL after HBOT is clearly demonstrated. Moreover, the analysis evidently demonstrates that the effect of HBOT on these “not completely blinded evolution” was the same in the treated group and the control group after blind randomization. The correlation between the improvement in NIHSS and ADL and the improvement in the brain SPECT results, which was done in a completely blinded fashion, further substantiates the clinical findings. Moreover, the consistency between the anatomical locations of the changes in the brain metabolism, as demonstrated by the SPECT, with the finding in the neurological evaluation provides important validation of the neurological evaluation.

During most of the 20^th^ century, there was an ongoing debate about the time window available for induction of neuroplasticity. The improvements in the chronic late stage reported here support the view that neuroplasticity can be activated months to years after the acute event when a proper brain stimulation (such as HBOT) is applied. More specifically, the current study included patients that underwent stroke more than 6 months prior to treatment and after their condition reached a steady state (no improvements were monitored for at least a month). These important and unexpected findings arein agreement with other recent findings revealing that many aspects of the brain remain plastic even at adulthood [38]. They are also consistent with several other studies in post stroke patients [39], [40], [41]. In the current study, patients were treated only with HBOT without any additional guided training and/or practice. This was done in order to demonstrate the therapeutic potential of this treatment. It is reasonable to expect that exploiting the HBOT in conjunction with other rehabilitation intervention can lead to even better results leading to optimal future practice. The current study paved the way for future investigations of this promising direction, which should be one of the aims of upcoming more elaborated clinical studies.

Current imaging technologies reveal that the stunned brain areas (regions of high anatomy-physiology mismatch) may persist for months and years after an acute brain event [11], [12], [13]. The changes in SPECT images after treatment demonstrate that the HBOT procedure led to reactivations of neuronal activity in the stunned areas. While SPECT imaging has limited spatial resolutions (in comparison, for example, to fMRI), the changes in activity were sufficiently robust to be clearly detected by the SPECT images. However, a future, more detailed study using fMRI (along with direct observations on animal model) will be able to provide additional valuable insights, in particular regarding the operative underlying mechanisms that activate the neuroplasticity (e.g. the putative role of glial cells). We note that patients were not selected based on their anatomical and functional brain imaging evaluation. It might be possible that the results would have been even better had the study included only patients with high SPECT/CT mismatch. This issue of the preferred population for HBOT should be further investigated in future clinical trials. We also note that in the current pioneering study aimed at “proof of concept”, all patients underwent 40 HBOT sessions. Based on our current clinical experience, more sessions of HBOT may be needed, at least for some patients, in order to obtain the maximal improvement effect.

In any case, the observed reactivation of neuronal activity in the stunned areas imply that increasing the plasma oxygen concentration with hyperbaric oxygenation is a potent means of delivering to the brain sufficient oxygen for tissue repair: HBOT might initiate a cellular and vascular repair mechanism and improve cerebral vascular flow [8], [13], [16], [17]. At the cellular level, HBOT can improve mitochondrial function (in both neurons and glial cells) and cellular metabolism; improve BBB and inflammatory reactions; reduce apoptosis; alleviate oxidative stress; increase levels of neurotrophins and nitric oxide, and up-regulate axon guidance agents [13], [16], [17], [20]. Moreover, the effects of HBOT on neurons can be mediated indirectly by glial cells, including asrocytes [18]. HBOT may also promote neurogenesis of the endogenous neural stem cells [19]. The major limitation of the above-mentioned data is that it has been tested in different types of models and includes different protocols of HBOT. However, it is well noticed that there is at least one common denominator to all repair/regeneration mechanisms: they are all energy/oxygen dependent. It might be possible that HBOT enables the metabolic change simply by supplying the missing energy/oxygen needed for those regeneration processes.

To conclude, in this study we provide, for the first time, convincing results demonstrating that HBOT can induce significant neurological improvement in post stroke patients. The neurological improvements in a chronic late stage demonstrate that neuroplasticity can be operative and activated by HBOT even long after acute brain insult. Thus, the findings have important implications that can be of general relevance and interest in neurobiology. Although this study focused on stroke patients, the findings bear the promise that HBOT may serve as a valuable therapeutic practice in other neurological disorders exhibiting discrepancy between the anatomical and functional evaluation of the brain.

## Supporting Information

Protocol S1 Clinical Study Protocol.
(DOCX)

Checklist S1 CONSORT 2010 checklist.
(DOCX)

Form S1 Informed consent form (English translation).
(PDF)

Text S1 Statistical, Randomization and Placebo Considerations.
(DOCX)

Text S2 Scatter plot analysis of the clinical scores.
(DOCX)
