# Supplementary material for: Hyperbaric Oxygen Induces Late Neuroplasticity in Post Stroke Patients - Randomized, Prospective Trial
Source: PLoS One. 2013 Jan 15;8(1):e53716. doi: 10.1371/journal.pone.0053716 (PMC3546039; doi:10.1371/journal.pone.0053716)
Supplement: Protocol S1 — Clinical Study Protocol. (DOCX) [file pone.0053716.s001.docx]

S1: Clinical Study Protocol

| Study Title: | **Hyperbaric Oxygen Therapy Induced Neuroplasticity in Post Stroke Patients Suffering Chronic Neurological Deficiencies.** |
| --- | --- |
|  |  |
|  |  |
|  |  |
| Protocol Number:  Investigational Product: | HBOT-CVA -01 Version 3.0    Hyperbaric Oxygen Therapy, 2ATM, 100% oxygen  Hyperbaric chamber, Multiplace at Assaf-Harofeh Medical Center, Israel |
|  |  |
| Primary Investigator:  Study Director: | Dr. Shai Efrati  Fax: +972-(0)8-920-4989  Phone: +972-(0)8-977-9393/5  Cell : +972-549-212-866  Email : [efratishai@013.net](mailto:efratishai@013.net)  Yair Bechor Fax: +972-(0)8-920-4989  Cell: +972-577-345-624 |
|  |  |
|  |  |

**Content**

**Pages**

Background………………………………………………………………………………....3-7

Objectives…………………………………………………………………………………....7

Study design……………………………………………………………………………......7-8

Inclusion criteria……………………………………………………………………..8

Exclusion criteria…………………………………………………………………..8-9

Study protocol……………………………………………………………………....9

Neurological evaluation ………………………………………………………………….10-12

National Institutes of Health Stroke Scale (NIHSS)……………………………….10

Activities of daily living (ADL)…………………………………………………....10

Brain functional Imaging……………………………………………………….…10-12

Quality of life ……………………………………………………………………....12

Administration and regulation……………………………………………………………...13

Informed Consent…………………………………………………………………..13

Confidentiality ……………………………………………………………………..13

Study Files……………………………………………………………………….…13

Statistical considerations...……………………………………………………………...14-15

Safety Analysis Set………………………………………………………….……..14

Primary Efficacy Analysis Set……………………………………………………..14

Sample Size Considerations………………………………………………….…....14

Primary Efficacy Analysis……………………………………………………….14-15

Randomization ……………………………………………………………………….…..15-16

Adverse Events……………………………………………………………………………16-20

References ………………………………………………………………………………..21-25

Appendix 1. National Institutes of Health Stroke Scale (NIHSS)………………………..26-29

Appendix 2. Activities of daily living (ADL) questioner …………………………………30

Appendix 3. EQ-5D questioner …………………………………………………………31-32

**Background**

Stroke is one of the leading causes of death and disabilities in elderly population worldwide. In high-income countries, stroke is the third most common cause of death and the main cause of acquired adult disability [^1^](#_ENREF_1). The most widespread and widely recognized mutilation caused by stroke is motor impairment, although other cognitive functions may also be impaired ^[1-3](#_ENREF_1)^. Intensive functional therapy and rehabilitation programs are essential to minimize the cognitive and physical sequels associated with acquired brain injury, in order to maximize the patient's quality of life [^4^](#_ENREF_4)^,^ [^5^](#_ENREF_5). Unfortunately, these programs are only partially successful, and alternative approaches are needed to expand metabolic recovery of the injured cerebral tissues. Hyperbaric oxygen therapy (HBOT) has been proposed as a possible post-stroke adjuvant therapy. However, the results of HBOT application during the acute phase, i.e. immediately after stroke, are contradictory ^[6-9](#_ENREF_6)^, whereas data on the effect of HBOT in the chronic stage are scanty.

HBOT has been investigated for treatment of numerous diseases for more than 300 years. The principal effect of HBOT is increasing the solubility of oxygen in plasma to a level sufficient to support tissues with minimal oxygen supply carried on by hemoglobin. Transport of oxygen to mitochondria, the main sites of oxygen utilization within each individual cell, occurs by diffusion, via a stepwise decrease in the driving oxygen pressure gradient. Diffusion oxygen gradient is a [vector](http://en.wikipedia.org/wiki/Vector_field)  indicating the direction of the greatest rate of change between oxygen dissolved in the blood and oxygen within the cell/entire tissue. As a whole, breathing oxygen under hyperbaric conditions has been shown to be a potent means of increasing arterial oxygen tension, as well as brain oxygen tension [^10^](#_ENREF_10)^,^ [^11^](#_ENREF_11) . For example, at 2 absolute atmospheres (ATA), plasma O_2_ tension rises above 1110 mmHg, whereas at normal environmental conditions, i.e. at the sea level, it reaches only 98 mmHg. As can be concluded, hyperbaric conditions can provide about a ten-fold increase in the amounts of O_2_ reaching the hypoxic brain tissue. HBO therapy is well tolerated and has been considered safe when used according to the standard protocols, with oxygen pressure not exceeding 3 ATA and treatment sessions limited to a maximum of 120 min [^12^](#_ENREF_12).

It has been speculated that following hypoxic/ischemic episodes, mitochondrial membrane alterations, microvascular failure and intermittent cerebral blood flow (CBF) reduction might impair efflux of oxygen from the capillaries into the tissue, and subsequently - into the neurons and then into the mitochondria, thus contributing to the functional failure of aerobic metabolism [^13^](#_ENREF_13) and development of oxygen delivery/demand mismatch. Increasing plasma oxygen concentrations by means of HBOT is a potent tool for urgent oxygen supply to the perfused tissue ^[14](#_ENREF_14)^. Moreover, HBOT might augment oxygen delivery to the hypoxic brain areas by yet a different mechanism, namely via increasing deformability of the red blood cells [^15^](#_ENREF_15). Under normal conditions, HBOT application to the preserved, uninjured brain tissue also enhances brain oxygenation, albeit concomitantly induces vasoconstriction [^16^](#_ENREF_16). It has been demonstrated, both in experimental [^17-19^](#_ENREF_17) and clinical studies [^20^](#_ENREF_20)^,^ [^21^](#_ENREF_21), that CBF of injured brain does, indeed, decrease following exposure to HBOT. Furthermore, a propensity for vasoconstriction and decrease in CBF during exposure to HBOT was observed in uninjured areas of the brain, whereas in the injured areas CBF showed a tendency to increase ^[22](#_ENREF_22)^. Therefore, it can be suggested that HBOT of the same magnitude differentially and selectively affects blood vessels within different brain areas, depending on the severity of the injury ^[22-24](#_ENREF_22)^. It has been suggested that by such mechanism HBOT might initiate and/or regulate cellular and vascular repair processes within the injured brain [^7^](#_ENREF_7)^,^ [^25^](#_ENREF_25). Indeed, HBOT was shown to improve CBF in patients with chronic neurological deficiency, as demonstrated by brain SPECT imaging [^26^](#_ENREF_26)^,^ [^27^](#_ENREF_27).

The effects of HBOT on the damaged/ischemic neuronal tissue has been extensively evaluated [^28^](#_ENREF_28). Among the most important findings, the following should be mentioned: recovery of mitochondrial membranes and their functioning [^29-33^](#_ENREF_29); improved BBB and decreased secondary inflammatory reactions [^34^](#_ENREF_34)^,^ [^35^](#_ENREF_35); improved cellular metabolism [^7^](#_ENREF_7)^,^ [^26^](#_ENREF_26)^,^ [^35-38^](#_ENREF_35); initiation of anti-inflammatory activities, such as downregulation of ICAM-1 and other inflammatory cytokines [^28^](#_ENREF_28)^,^ [^35^](#_ENREF_35)^,^ [^39^](#_ENREF_39)^,^ [^40^](#_ENREF_40), diminished infiltration and reduced adhesion of leucocytes within the injured brain [^35^](#_ENREF_35)^,^ [^41-43^](#_ENREF_41); reduced COX-2 expression [^44^](#_ENREF_44), decreased synthesis of apoptosis-regulating proteins, e.g. caspase 3, caspase 9, bcl-2 and p53 and, accordingly, reduced apoptosis [^35^](#_ENREF_35)^,^ [^45^](#_ENREF_45)^,^ [^46^](#_ENREF_46); reduced expression of HIF-1 [^47^](#_ENREF_47)^,^ [^48^](#_ENREF_48); increased Na+-K+ ATPase activity and ATP production, resulting in stabilization of transmembrane ion gradients [^49^](#_ENREF_49); upregulation of antioxidant enzyme synthesis and reduction of hydroxyl radical formation [^50-53^](#_ENREF_50); suppression of NADPH oxidase [^54^](#_ENREF_54) activity; increased expression of neurotrophin [^44^](#_ENREF_44)^,^ [^55^](#_ENREF_55); increased NO activity [^56^](#_ENREF_56); inhibited Nogo-A, Ng-R, or RhoA expression [^57^](#_ENREF_57); up-regulated presence of axon guidance agents [^35^](#_ENREF_35) .

HBOT-associated neuronal effects can be also mediated indirectly, via glial cells and asrocytes ^[58](#_ENREF_58)^. Glial cells, which are robustly activated after cerebral insult, are known to play important role in the processes associated with neuroprotection and/or neurodegeneration [^59^](#_ENREF_59)^,^ [^60^](#_ENREF_60). In a variety of pathologic conditions, including ischemia, microglial cells release soluble factors such as pro-inflammatory cytokines and proteases, which are directly linked to neurodegeneration [^61-63^](#_ENREF_61) By contrast, astrocyte activities promote the recovery of neuronal functions after injury by providing the energy substrates and trophic factors to btain cells, act as free radical and glutamate scavengers, promote remyelination and neovascularization and stimulate neurogenesis from neural stem cells [^64^](#_ENREF_64). In permanent cerebral ischemia, astrocytes might be involved in regulation of high-to-toxic ATP concentrations by the induced expression of specific P2X receptor subtypes ^[65](#_ENREF_65)^. In a model of permanent cerebral ischemia, HBOT has been demonstrated to decrease microgliosis, but to increase astrogliosis, within the peri-infarct areas ^[58](#_ENREF_58)^. HBOT treatment has been shown to promote neurogenesis of the endogenous neural stem cells, thus also contributing to the repair processes within the injured brain ^[66-68](#_ENREF_66)^.

The major limitation, interfering with any conclusions drawn from the information concerning the metabolic effects of HBOT at the cellular level, is that these conclusions would be based on a striking diversity among the experimental models, types of neurologic damage and varieties of HBOT protocols (with respect to the applied pressure and duration of treatments). Nevertheless, there exists at least one underlying common denominator: unexceptionally, all described repair/regeneration mechanisms are energy/oxygen dependent. Consequently, at least one conclusion is unequivocal: HBO therapy improves metabolic processes of the neuronal tissue by providing the energy/oxygen supply, mandatory for successful regeneration processes within the brain recuperating from severe injury.

The improved metabolic platform generated by HBOT is expected to initiate and enable many regeneration processes. In other organs suffering from oxygen supply/ demand mismatch, such as non-healing wounds in diabetic patients, it was already prove that HBOT can initiate many regeneration process eventually culminate in wound healing. The same is expected to happen in the relatively hypoxic-energy depleted areas of the injured brain.

**Objectives**

The aim of the current study is to evaluate, for the first time in a prospective randomized study, the effect of hyperbaric oxygen therapy (HBOT) on patients with **chronic** neurological deficiency due to stroke.

- The primary endpoints of the study are to evaluate the effects of the HBOT on:
- National Institutes of Health Stroke Scale (NIHSS)
- Activities of daily living (ADL)
- Brain metabolism as visualized by SPECT
- The secondary endpoints included quality of life evaluation.

The safety of the HBOT in this post stroke population will also be evaluated and any adverse event will be recorded.

**Study design**

A prospective, randomized, control-crossed over trial.

The study will be done in the hyperbaric chamber and in the research and development unit of Assaf Harofeh Medical Center, Israel.

The brain SPECT evaluation will be done in Nuclear Medicine institute in Assaf-Harofeh Medical Center, Israel.

**Inclusion criteria**

- Ischemic or hemorrhagic stroke 6-36 months prior to the inclusion in the study.
- All patients had to have persistent (stable non improving) neurologic deficiency that includes at least one motor dysfunction (paresis or plegia) without noticeable improvement during the last month prior to their enrolment.
- age 18 years or older

**Exclusion criteria**

Patients will be excluded if they will have one of the following criteria:

- Dynamic neurologic improvement or worsening during the last month
- Had been treated with HBOT for any other reason prior to their inclusion
- Have any other indication for HBOT;
- Chest pathology incompatible with pressure changes;
- Inner ear disease;
- Patients suffering from claustrophobia;
- Inability to sign written informed consent.
- Smoking patients were not allowed to smoke during the study and if they could not comply with this demand they were excluded.
- Inability to sign informed consent

***Study protocol***

After signing the informed consent, patients were randomized in 1:1 manner into the treated or the control-cross group. After the randomization, patients were invited for baseline evaluation that included full review of their medical status and complete physical examination. All patients had baseline CT scan. After their inclusion patients were randomized to two groups: a treated group and a cross group. All patients had evaluation of their neurologic functions by physical examination, ability to perform activities of daily living, quality of life and brain functional imaging (rCBF-SPECT scan). The patients in the treated group were evaluated twice – at baseline and after 2 months of HBOT treatment. The patients of the cross group were evaluated three times – at baseline, after a 2 month control period of no treatment and after a consequent 2 month period of HBOT treatment. We emphasize that the study was a cross-over trial and patients in the cross group received HBOT treatment after their second evaluation and had a third neurological evaluation after the cross, when they have completed their HBOT.

The following HBOT treatment protocol was practice: The patient were treated 40 times (each treatment session was given on a separate day) distributed over two months (five days a week). Each session was for 90 minutes in 100% oxygen atmosphere and at pressure of 2 ATA.

***Neurologic evaluation***

**National Institutes of Health Stroke Scale (NIHSS)**

The neurological evaluation will be done at baseline and after 2 months for all patients. In the cross group a 3^rd^ evaluation will be performed after 4 month- after crossing and completing 2 months of HBOT treatment. The clinical severity of the stroke will be assess by trained physician according to the National Institutes of Health Stroke Scale (NIHSS)[^69^](#_ENREF_69)^,^ [^70^](#_ENREF_70).

The NIHSS is attached in appendix 1.

**Activities of daily living (ADL)**

The activities of daily living (ADL) will be evaluated by a questioner that covers the following functions: bathing, dressing, grooming, oral care, toileting, walking, climbing stairs, eating, shopping, cooking, managing medications, using phone, housework, doing laundry, driving and managing finances[^71^](#_ENREF_71). For each criterion the patient had to define whether he/she is independent, needs help, dependent or does not do at all (The range of the total score is from 0 (best) to 51 (worst)).

The ADL is attached in appendix 2.

**Brain functional Imaging SPECT imaging and Analysis**

Single photon emission computed tomography (SPECT) will be conducted before and after HBOT. Subjects will lay supine in a quiet dimly lit room for 20 min prior to injection of the radiopharmaceutical. Apart from administration of the injection by a physician, they will remainalone in the room during this period. Subjects will be asked to remain at rest for 10 min after the injection of the radiopharmaceutical to allow uptake of the radiopharmaceutical in the brain.

An injection of 925-1110 MBq (25-30 mCi) of technetium-99m ethyl cysteinate dimmer (Tc-99m-ECD) will be given into an arm vein through a previously placed intravenous cannula. SPECT imaging of the brain was performed, at 40-60 min post injection, with the subject’s head supported by a headrest, using a dual detector gamma camera (ECAM or Symbia T, Siemens Medical Systems) equipped with high resolution collimators. Data will be acquired in the step-and-shoot mode, using a 360 degree circular orbit, with the detectors of the gamma camera as close as possible to the subject’s head. The camera used for imaging will noted for each subject and the same camera will be used for the follow-up study. Data will be acquired using a 128׳128 image matrix in 3 degree steps of 20 seconds per step. Data will be reconstructed by iterative reconstruction with no filtering. The Chang method (*μ*=0.12/ cm) will be used for attenuation correction.

Intra subject visual analysis will be conducted using a process in which pre and post treatment studies are fused and normalized to pre-treatment whole brain activity.

Visual analysis will be carried out by two nuclear medicine physicians who compared the scans independently and grad them as either: 1=no change, 2=mild change and 3=significant change. Where, no change is assign when no visual difference is noted in the number or size of perfusion deficits, mild change is given when a reduced number of perfusion defects are noted or the size of the perfusion defects is reduced. Significant change should be attributed when a global perfusion increment is observe in addition to diminution of defect numbers or size. Differences in evaluation will be resolve after reviewing the images together. Scan visual interpretation will be carried out while blinded to any laboratory or clinical data.

A comparison of the SPECT results with anatomical imaging (CT or MRI) will be done in order to evaluate the extent of perfusion deficit in relation to the anatomical lesion.

***Quality of life evaluation***

Quality of live will be evaluated by the EQ-5D questioner[^72^](#_ENREF_72)^,^ [^73^](#_ENREF_73). EQ-5D essentially consists of 2 pages - the EQ-5D descriptive system and the EQ visual analogue scale (EQ VAS). The EQ-5D descriptive system comprises the following 5 dimensions: mobility, self-care, usual activities, pain/discomfort and anxiety/depression. Each dimension has 3 levels: no problems, some problems, extreme problems.

The EQ VAS records the respondent’s self-rated health on a vertical, visual analogue scale where the endpoints are labeled “Best imaginable health state” and “Worst imaginable health state” (0 is denoting the worst imaginable health state while 100 is denoting the best imaginable health state). The validity and reliability of the EQ­5D questionnaire have been tested[^74^](#_ENREF_74)^,^[^75^](#_ENREF_75).

The EQ-5D questioner is attached in appendix 3.

**Administration and regulation**

**Informed Consent**

The investigator will obtain written informed consent from the patient participates in this study after adequate explanation of the aims, methods, objectives, and potential hazards of the study and prior to undertaking any study-related procedures. The investigator must utilize a consent form for documenting written informed consent. Informed consent will be appropriately signed and dated by the patient or the subject’s legally authorized representative and the person obtaining consent.

**Confidentiality**

Subjects’ anonymity will be strictly maintained and that their identities are protected from unauthorized parties. The information is not to be disclosed to any third party (except for medical stuff or employees or agents directly involved in the conduct of the study or as required by law).

**Study Files**

The medical records will be maintained adequately to enable good data storage and latter on management. Subject clinical source documents would include (although not, limited to) the following: subject hospital/clinic/ hyperbaric unit records, physician’s and nurse’s notes, appointment book, original laboratory reports, electroencephalogram (EEG), X-ray, SPECTs, CT and special assessment reports, consultant letters, screening and enrollment log etc.

**Statistical Considerations**

## Analysis Sets

### Safety Analysis Set

The safety analysis set will consist of all subjects for whom the study treatment was initiated.

### Primary Efficacy Analysis Set

In this trial will be measured the co-primary endpoint parameters: *improvement in NIHSS tests score* and *improvement in ADL score* following HBOT treatment for post stroke patients suffering chronic neurological deficiencies.

The primary efficacy analysis evaluation will include all subjects who completed the HBOT treatment or the control period; had no major protocol violations, and for whom there is a valid *NIHSS and ADL tests score* following 2 months of HBOT treatment.

**Sample Size Considerations**

Presentation of sample size is based on achieving 80% power overall to demonstrate that improvement rate in *NIHSS test score* is at least 0.25 and, separately, that improvement rate in *ADL test score* is at least 0.25. An improvement rate of at least 25% in the NIHSS test and ADL score in the treatment group (comparing to baseline, prior to HBOT treatment) would be an appropriate clinical target for HBOT treatment success and of sufficient interest to encourage further investigation of HBOT treatment for post stroke patients suffering chronic NIHSS deficiencies.

Sample size was based on the assumption that exposure to the NIHSS tests and ADL evaluation (at baseline) without any additional training might induce up to 4% score improvement in the second NIHSS test (following treatment). Assuming a true success rate of 25% a sample of N = 31 will provide 80% power to show that HBOT treatment induces at least 25% improvement on *NIHSS test score* and separately, on *ADL test score*. This is based on a power analysis using the normal approximation for the binomial, with one-sided Alpha=0.05.

## Statistical Analysis

### Safety Analysis

AE's will be tabulated by treatment group, severity and relation to treatment.

### Primary Efficacy Analysis

This study has co-primary endpoints: *NIHSS tests score* and *ADL score.* For each of the co-primary endpoints we will compute Overall Proportion of Success:

$$\text{P(success) }=\text{ }\frac{\text{Total \# of Successes}}{\text{Total \# in Cohort}}$$

The following hypotheses are specified and will be tested separately:

For *NIHSS tests score* proportion of improvement:

H_0_: p_1_< p _2_

H_1_: p _1_> p _2_

While p1= 0.25 and p2=0.04

For *ADL score* proportion of improvement:

H_0_: p_1_< p _2_

H_1_: p _1_> p _2_

While p1= 0.25 and p2=0.04

Each of these hypotheses will be tested by:

1. Constructing a one-sided, lower 95% confidence interval about the observed Overall Proportion of Success in the relevant cohort.
2. Examining whether the lower limit of the confidence interval is at or above the success criterion

Study success will be declared if the following is met:

1. Lower confidence limit of HBOT treatment success is at 0.25 or above

**Randomization**

Since the diversity of the patients included in the study, after signing the informed consent was expected to be high, no stratifies criteria was used. Patients were randomized in 1:1 fashion to either treatment or controlled-cross group.

There is no placebo in the cross group during the control period. The only way to give “placebo” of HBOT, is to bring the patients to the hyperbaric chamber and to increase the environmental pressure- in a way the patients will “feel the hyperbaric pressure” in their ears. However, Henry’s law states: “the amount of a given gas dissolved in a given type and volume of liquid is directly proportional to the pressure of that gas in equilibrium with that liquid”. Thus, hyperbaric environment significantly increases the dissolved oxygen pressure even if a person holding his breath[^76^](#_ENREF_76). Moreover, the alternative of hosting the patients in the hyperbaric chamber without any pressure increase cannot serve as a real placebo since the patients will not feel the pressure. Accordingly, the only real “placebo” could be archived by increasing the pressure and reducing the percentages of the oxygen but that holds significant safety consideration.

From the ethical point of view, it was deemed unethical to “treat” the patients (40 sessions in the hyperbaric chamber) while they actually know that it is a placebo. In order to somewhat compensate for this limitation, the control was a cross group and after the 2^nd^ evaluation at the end of a control period they were crossed to HBOT. The cross for treatment enables intra-group efficacy evaluation in both treatment and control group.

Since the patients knew they were not receiving HBOT during the control period they were not blinded with regard to the treatment arm. Accordingly the ADL and quality of life evaluations were not blinded. Moreover, in the current clinical setting, it was not possible to assure that the NIHSS evaluation could be done completely blinded by the physician. The complete blindness evaluation could be assured in the brain SPECT evaluation done by the radiologist. The correlation between metabolic blinded evaluation of the brain (SPECT) and the neurological un-blinded evaluations give further support and strength to the clinical findings.

**Adverse Events**

An adverse event (AE) is any untoward medical occurrence in a clinical investigation subject administered a medicinal product and which does not necessarily have a causal relationship with this treatment. An AE can therefore be any unfavorable and unintended sign, symptom, or disease temporally associated with the use of a medicinal product, whether or not considered related to the medicinal product. Pre-existing events, which increase in severity or change in nature during or as a consequence of use of a medicinal product in human clinical trials, will also be considered AEs.

Any medical condition or clinically significant laboratory abnormality with an onset date before the screening visit and not related to study procedures is considered to be pre-existing, and should be documented in the case report form.

Any AE (i.e., a new event or an exacerbation of a pre-existing condition) with an onset date after the screening visit up to the last day on study (including the follow-up, off study medication period of the study), should be recorded as an AE on the appropriate CRF page(s).

An AE does not include:

- Medical or surgical procedures (e.g. surgery, Endoscopy, tooth extraction, transfusion); the condition that leads to the procedure are an adverse event.
- Pre-existing diseases or conditions or laboratory abnormalities present or detected prior to the screening visit that does not worsen.
- Situations where an untoward medical occurrence has not occurred (e.g. hospitalization for elective surgery, social and/or convenience admissions).
- Overdose of either study drug or concomitant medication without any signs or symptoms unless the subject is hospitalized for observation.

***Assessment of Adverse Events***

All AEs will be assessed by the investigator and recorded on the appropriate CRF page, including the date of onset and resolution, severity, relationship to study drug or study procedures, outcome and action taken with study medication.

The relationship to study drug therapy or study procedures should be assessed using the following definitions:

**No**: Evidence exists that the adverse event has an etiology other than the study drug or study procedures (e.g. pre-existing condition, underlying disease, intercurrent illness, or concomitant medication).

**Yes**: A temporal relationship exists between the event onset and administration of the study drug or between the event and the study procedures. It cannot be readily explained by the subject’s clinical state or concomitant therapies and, in the case of the study drug, appears with some degree of certainty to be related based on the known therapeutic and pharmacologic actions or adverse event profile of the study drug. In case of cessation or reduction of the dose, the event abates or resolves and reappears upon re-challenge. It should be emphasized that ineffective treatment should not be considered as causally related in the context of adverse event reporting.

These criteria in addition to good clinical judgment should be used as a guide for determining the causal assessment. If it is felt that the event is not related to study drug therapy, then an alternative explanation should be provided.

***Serious Adverse Events***

**A** **serious adverse event** (SAE) is defined as follows:

Any adverse drug experience occurring at any dose that results in any of the following outcomes:

- Death;
- Life-threatening situation (subject is at **immediate** risk of death);
- In-patient hospitalization or prolongation of existing hospitalization (excluding those for study therapy or placement of an indwelling catheter, unless associated with other serious events);
- Persistent or significant disability/incapacity;
- Congenital anomaly/birth defect in the offspring of a subject who received study drug;

Other: medically significant events that may not result in death, be immediately life-threatening, or require hospitalization, may be considered a SAE when, based upon appropriate medical judgment, they may jeopardize the Subject and may require medical or surgical intervention to prevent one of the outcomes listed in this definition.

Examples of such events are:

- Intensive treatment in an emergency room
- Blood dyscrasias or convulsions that do not result in hospitalization
- Development of drug dependency or drug abuse

The investigator should notify the Institutional Review Board (IRB) or Independent Ethics Committee (IEC) as soon as is practical, of serious events in writing where this is required by local regulatory authorities, and in accordance with the local institutional policy.

**References**

1. Langhorne P, Coupar F, Pollock A. Motor recovery after stroke: a systematic review. Lancet Neurol. 2009; **8**(8): 741-54.

2. Carod-Artal FJ, Egido JA. Quality of life after stroke: the importance of a good recovery. Cerebrovasc Dis. 2009; **27 Suppl 1**: 204-14.

3. Lim C, Alexander MP. Stroke and episodic memory disorders. Neuropsychologia. 2009; **47**(14): 3045-58.

4. Teasell R. Forward. An international perspective of stroke rehabilitation. Top Stroke Rehabil. 2009; **16**(1): v.

5. Prvu Bettger JA, Stineman MG. Effectiveness of multidisciplinary rehabilitation services in postacute care: state-of-the-science. A review. Arch Phys Med Rehabil. 2007; **88**(11): 1526-34.

6. Anderson DC, Bottini AG, Jagiella WM, Westphal B, Ford S, Rockswold GL, et al. A pilot study of hyperbaric oxygen in the treatment of human stroke. Stroke. 1991; **22**(9): 1137-42.

7. Zhang JH, Lo T, Mychaskiw G, Colohan A. Mechanisms of hyperbaric oxygen and neuroprotection in stroke. Pathophysiology. 2005; **12**(1): 63-77.

8. Poli S, Veltkamp R. Oxygen therapy in acute ischemic stroke - experimental efficacy and molecular mechanisms. Curr Mol Med. 2009; **9**(2): 227-41.

9. Helms AK, Whelan HT, Torbey MT. Hyperbaric oxygen therapy of cerebral ischemia. Cerebrovasc Dis. 2005; **20**(6): 417-26.

10. Niklas A, Brock D, Schober R, Schulz A, Schneider D. Continuous measurements of cerebral tissue oxygen pressure during hyperbaric oxygenation--HBO effects on brain edema and necrosis after severe brain trauma in rabbits. J Neurol Sci. 2004; **219**(1-2): 77-82.

11. Reinert M, Barth A, Rothen HU, Schaller B, Takala J, Seiler RW. Effects of cerebral perfusion pressure and increased fraction of inspired oxygen on brain tissue oxygen, lactate and glucose in patients with severe head injury. Acta Neurochir (Wien). 2003; **145**(5): 341-9; discussion 9-50.

12. Tibbles PM, Edelsberg JS. Hyperbaric-oxygen therapy. N Engl J Med. 1996; **334**(25): 1642-8.

13. Calvert JW, Cahill J, Zhang JH. Hyperbaric oxygen and cerebral physiology. Neurol Res. 2007; **29**(2): 132-41.

14. Veltkamp R, Warner DS, Domoki F, Brinkhous AD, Toole JF, Busija DW. Hyperbaric oxygen decreases infarct size and behavioral deficit after transient focal cerebral ischemia in rats. Brain Res. 2000; **853**(1): 68-73.

15. Demchenko IT, Luchakov YI, Moskvin AN, Gutsaeva DR, Allen BW, Thalmann ED, et al. Cerebral blood flow and brain oxygenation in rats breathing oxygen under pressure. J Cereb Blood Flow Metab. 2005; **25**(10): 1288-300.

16. Zhilyaev SY, Moskvin AN, Platonova TF, Gutsaeva DR, Churilina IV, Demchenko IT. Hyperoxic vasoconstriction in the brain is mediated by inactivation of nitric oxide by superoxide anions. Neurosci Behav Physiol. 2003; **33**(8): 783-7.

17. Rosenthal RE, Silbergleit R, Hof PR, Haywood Y, Fiskum G. Hyperbaric oxygen reduces neuronal death and improves neurological outcome after canine cardiac arrest. Stroke. 2003; **34**(5): 1311-6.

18. Mink RB, Dutka AJ. Hyperbaric oxygen after global cerebral ischemia in rabbits does not promote brain lipid peroxidation. Crit Care Med. 1995; **23**(8): 1398-404.

19. Schabitz WR, Schade H, Heiland S, Kollmar R, Bardutzky J, Henninger N, et al. Neuroprotection by hyperbaric oxygenation after experimental focal cerebral ischemia monitored by MRI. Stroke. 2004; **35**(5): 1175-9.

20. Rockswold SB, Rockswold GL, Vargo JM, Erickson CA, Sutton RL, Bergman TA, et al. Effects of hyperbaric oxygenation therapy on cerebral metabolism and intracranial pressure in severely brain injured patients. J Neurosurg. 2001; **94**(3): 403-11.

21. Sukoff MH, Ragatz RE. Hyperbaric oxygenation for the treatment of acute cerebral edema. Neurosurgery. 1982; **10**(1): 29-38.

22. Bergo GW, Engelsen B, Tyssebotn I. Unilateral frontal decortication changes cerebral blood flow distribution during hyperbaric oxygen exposure in rats. Aviat Space Environ Med. 1993; **64**(11): 1023-31.

23. Kanai N, Hayakawa T, Mogami H. Blood flow changes in carotid and vertebral arteries by hyperbaric oxygenation. Neurology. 1973; **23**(2): 159-63.

24. Rogatsky GG, Shifrin EG, Mayevsky A. Physiologic and biochemical monitoring during hyperbaric oxygenation: a review. Undersea Hyperb Med. 1999; **26**(2): 111-22.

25. Kuffler DP. Hyperbaric oxygen therapy: an overview. J Wound Care. **19**(2): 77-9.

26. Golden ZL, Neubauer R, Golden CJ, Greene L, Marsh J, Mleko A. Improvement in cerebral metabolism in chronic brain injury after hyperbaric oxygen therapy. Int J Neurosci. 2002; **112**(2): 119-31.

27. Neubauer RA, James P. Cerebral oxygenation and the recoverable brain. Neurol Res. 1998; **20 Suppl 1**: S33-6.

28. Fischer BR, Palkovic S, Holling M, Wolfer J, Wassmann H. Rationale of hyperbaric oxygenation in cerebral vascular insult. Curr Vasc Pharmacol. **8**(1): 35-43.

29. Daugherty WP, Levasseur JE, Sun D, Rockswold GL, Bullock MR. Effects of hyperbaric oxygen therapy on cerebral oxygenation and mitochondrial function following moderate lateral fluid-percussion injury in rats. J Neurosurg. 2004; **101**(3): 499-504.

30. Zhou Z, Daugherty WP, Sun D, Levasseur JE, Altememi N, Hamm RJ, et al. Protection of mitochondrial function and improvement in cognitive recovery in rats treated with hyperbaric oxygen following lateral fluid-percussion injury. J Neurosurg. 2007; **106**(4): 687-94.

31. Palzur E, Zaaroor M, Vlodavsky E, Milman F, Soustiel JF. Neuroprotective effect of hyperbaric oxygen therapy in brain injury is mediated by preservation of mitochondrial membrane properties. Brain Res. 2008; **1221**: 126-33.

32. Gutsaeva DR, Suliman HB, Carraway MS, Demchenko IT, Piantadosi CA. Oxygen-induced mitochondrial biogenesis in the rat hippocampus. Neuroscience. 2006; **137**(2): 493-504.

33. Lou M, Chen Y, Ding M, Eschenfelder CC, Deuschl G. Involvement of the mitochondrial ATP-sensitive potassium channel in the neuroprotective effect of hyperbaric oxygenation after cerebral ischemia. Brain Res Bull. 2006; **69**(2): 109-16.

34. Veltkamp R, Siebing DA, Sun L, Heiland S, Bieber K, Marti HH, et al. Hyperbaric oxygen reduces blood-brain barrier damage and edema after transient focal cerebral ischemia. Stroke. 2005; **36**(8): 1679-83.

35. Rink C, Roy S, Khan M, Ananth P, Kuppusamy P, Sen CK, et al. Oxygen-sensitive outcomes and gene expression in acute ischemic stroke. J Cereb Blood Flow Metab. **30**(7): 1275-87.

36. Badr AE, Yin W, Mychaskiw G, Zhang JH. Effect of hyperbaric oxygen on striatal metabolites: a microdialysis study in awake freely moving rats after MCA occlusion. Brain Res. 2001; **916**(1-2): 85-90.

37. Lee JI, Wittsack HJ, Christaras A, Miese FR, Siebler M. Normalization of brain tissue lactate after hyperbaric oxygen therapy in a progressive stroke patient. Cerebrovasc Dis. 2008; **26**(4): 447-8.

38. Lou M, Zhang H, Wang J, Wen SQ, Tang ZQ, Chen YZ, et al. Hyperbaric oxygen treatment attenuated the decrease in regional glucose metabolism of rats subjected to focal cerebral ischemia: a high resolution positron emission tomography study. Neuroscience. 2007; **146**(2): 555-61.

39. Buras JA, Stahl GL, Svoboda KK, Reenstra WR. Hyperbaric oxygen downregulates ICAM-1 expression induced by hypoxia and hypoglycemia: the role of NOS. Am J Physiol Cell Physiol. 2000; **278**(2): C292-302.

40. Weisz G, Lavy A, Adir Y, Melamed Y, Rubin D, Eidelman S, et al. Modification of in vivo and in vitro TNF-alpha, IL-1, and IL-6 secretion by circulating monocytes during hyperbaric oxygen treatment in patients with perianal Crohn's disease. J Clin Immunol. 1997; **17**(2): 154-9.

41. Buras JA, Reenstra WR. Endothelial-neutrophil interactions during ischemia and reperfusion injury: basic mechanisms of hyperbaric oxygen. Neurol Res. 2007; **29**(2): 127-31.

42. Chen Q, Banick PD, Thom SR. Functional inhibition of rat polymorphonuclear leukocyte B2 integrins by hyperbaric oxygen is associated with impaired cGMP synthesis. J Pharmacol Exp Ther. 1996; **276**(3): 929-33.

43. Miljkovic-Lolic M, Silbergleit R, Fiskum G, Rosenthal RE. Neuroprotective effects of hyperbaric oxygen treatment in experimental focal cerebral ischemia are associated with reduced brain leukocyte myeloperoxidase activity. Brain Res. 2003; **971**(1): 90-4.

44. Yin W, Badr AE, Mychaskiw G, Zhang JH. Down regulation of COX-2 is involved in hyperbaric oxygen treatment in a rat transient focal cerebral ischemia model. Brain Res. 2002; **926**(1-2): 165-71.

45. Li Y, Zhou C, Calvert JW, Colohan AR, Zhang JH. Multiple effects of hyperbaric oxygen on the expression of HIF-1 alpha and apoptotic genes in a global ischemia-hypotension rat model. Exp Neurol. 2005; **191**(1): 198-210.

46. Vlodavsky E, Palzur E, Feinsod M, Soustiel JF. Evaluation of the apoptosis-related proteins of the BCL-2 family in the traumatic penumbra area of the rat model of cerebral contusion, treated by hyperbaric oxygen therapy: a quantitative immunohistochemical study. Acta Neuropathol. 2005; **110**(2): 120-6.

47. Sun L, Marti HH, Veltkamp R. Hyperbaric oxygen reduces tissue hypoxia and hypoxia-inducible factor-1 alpha expression in focal cerebral ischemia. Stroke. 2008; **39**(3): 1000-6.

48. Huang ZX, Kang ZM, Gu GJ, Peng GN, Yun L, Tao HY, et al. Therapeutic effects of hyperbaric oxygen in a rat model of endothelin-1-induced focal cerebral ischemia. Brain Res. 2007; **1153**: 204-13.

49. Mrsic-Pelcic J, Pelcic G, Vitezic D, Antoncic I, Filipovic T, Simonic A, et al. Hyperbaric oxygen treatment: the influence on the hippocampal superoxide dismutase and Na+,K+-ATPase activities in global cerebral ischemia-exposed rats. Neurochem Int. 2004; **44**(8): 585-94.

50. Freiberger J, Coulombe K, Suliman H, Carraway M, Piantadosi C. Superoxide dismutase responds to hyperoxia in rat hippocampus. Undersea Hyperb Med. 2004; **31**(2): 227-32.

51. Wada K, Miyazawa T, Nomura N, Tsuzuki N, Nawashiro H, Shima K. Preferential conditions for and possible mechanisms of induction of ischemic tolerance by repeated hyperbaric oxygenation in gerbil hippocampus. Neurosurgery. 2001; **49**(1): 160-6; discussion 6-7.

52. Wada K, Miyazawa T, Nomura N, Yano A, Tsuzuki N, Nawashiro H, et al. Mn-SOD and Bcl-2 expression after repeated hyperbaric oxygenation. Acta Neurochir Suppl. 2000; **76**: 285-90.

53. Yang ZJ, Xie Y, Bosco GM, Chen C, Camporesi EM. Hyperbaric oxygenation alleviates MCAO-induced brain injury and reduces hydroxyl radical formation and glutamate release. Eur J Appl Physiol. **108**(3): 513-22.

54. Ostrowski RP, Tang J, Zhang JH. Hyperbaric oxygen suppresses NADPH oxidase in a rat subarachnoid hemorrhage model. Stroke. 2006; **37**(5): 1314-8.

55. Yang JT, Chang CN, Lee TH, Lin TN, Hsu JC, Hsu YH, et al. Hyperbaric oxygen treatment decreases post-ischemic neurotrophin-3 mRNA down-regulation in the rat hippocampus. Neuroreport. 2001; **12**(16): 3589-92.

56. Ohgami Y, Chung E, Shirachi DY, Quock RM. The effect of hyperbaric oxygen on regional brain and spinal cord levels of nitric oxide metabolites in rat. Brain Res Bull. 2008; **75**(5): 668-73.

57. Zhou C, Li Y, Nanda A, Zhang JH. HBO suppresses Nogo-A, Ng-R, or RhoA expression in the cerebral cortex after global ischemia. Biochem Biophys Res Commun. 2003; **309**(2): 368-76.

58. Gunther A, Kuppers-Tiedt L, Schneider PM, Kunert I, Berrouschot J, Schneider D, et al. Reduced infarct volume and differential effects on glial cell activation after hyperbaric oxygen treatment in rat permanent focal cerebral ischaemia. Eur J Neurosci. 2005; **21**(11): 3189-94.

59. Morioka T, Kalehua AN, Streit WJ. Characterization of microglial reaction after middle cerebral artery occlusion in rat brain. J Comp Neurol. 1993; **327**(1): 123-32.

60. Kitamura Y, Nomura Y. Stress proteins and glial functions: possible therapeutic targets for neurodegenerative disorders. Pharmacol Ther. 2003; **97**(1): 35-53.

61. Kim NG, Lee H, Son E, Kwon OY, Park JY, Park JH, et al. Hypoxic induction of caspase-11/caspase-1/interleukin-1beta in brain microglia. Brain Res Mol Brain Res. 2003; **114**(2): 107-14.

62. Liu B, Hong JS. Role of microglia in inflammation-mediated neurodegenerative diseases: mechanisms and strategies for therapeutic intervention. J Pharmacol Exp Ther. 2003; **304**(1): 1-7.

63. Nakanishi H. Microglial functions and proteases. Mol Neurobiol. 2003; **27**(2): 163-76.

64. Liberto CM, Albrecht PJ, Herx LM, Yong VW, Levison SW. Pro-regenerative properties of cytokine-activated astrocytes. J Neurochem. 2004; **89**(5): 1092-100.

65. Franke H, Gunther A, Grosche J, Schmidt R, Rossner S, Reinhardt R, et al. P2X7 receptor expression after ischemia in the cerebral cortex of rats. J Neuropathol Exp Neurol. 2004; **63**(7): 686-99.

66. Wang XL, Yang YJ, Xie M, Yu XH, Liu CT, Wang X. Proliferation of neural stem cells correlates with Wnt-3 protein in hypoxic-ischemic neonate rats after hyperbaric oxygen therapy. Neuroreport. 2007; **18**(16): 1753-6.

67. Yang YJ, Wang XL, Yu XH, Wang X, Xie M, Liu CT. Hyperbaric oxygen induces endogenous neural stem cells to proliferate and differentiate in hypoxic-ischemic brain damage in neonatal rats. Undersea Hyperb Med. 2008; **35**(2): 113-29.

68. Wang XL, Yang YJ, Xie M, Yu XH, Wang QH. [Hyperbaric oxygen promotes the migration and differentiation of endogenous neural stem cells in neonatal rats with hypoxic-ischemic brain damage]. Zhongguo Dang Dai Er Ke Za Zhi. 2009; **11**(9): 749-52.

69. Kasner SE, Chalela JA, Luciano JM, Cucchiara BL, Raps EC, McGarvey ML, et al. Reliability and validity of estimating the NIH stroke scale score from medical records. Stroke. 1999; **30**(8): 1534-7.

70. Brott T, Adams HP, Jr., Olinger CP, Marler JR, Barsan WG, Biller J, et al. Measurements of acute cerebral infarction: a clinical examination scale. Stroke. 1989; **20**(7): 864-70.

71. Stefovska VG, Uckermann O, Czuczwar M, Smitka M, Czuczwar P, Kis J, et al. Sedative and anticonvulsant drugs suppress postnatal neurogenesis. Ann Neurol. 2008; **64**(4): 434-45.

72. Kuner R, Groom AJ, Muller G, Kornau HC, Stefovska V, Bresink I, et al. Mechanisms of disease: motoneuron disease aggravated by transgenic expression of a functionally modified AMPA receptor subunit. Ann N Y Acad Sci. 2005; **1053**: 269-86.

73. EuroQol--a new facility for the measurement of health-related quality of life. The EuroQol Group. Health Policy. 1990; **16**(3): 199-208.

74. Brazier J, Jones N, Kind P. Testing the validity of the Euroqol and comparing it with the SF-36 health survey questionnaire. Qual Life Res. 1993; **2**(3): 169-80.

75. van Agt HM, Essink-Bot ML, Krabbe PF, Bonsel GJ. Test-retest reliability of health state valuations collected with the EuroQol questionnaire. Soc Sci Med. 1994; **39**(11): 1537-44.

76. Muth CM, Radermacher P, Pittner A, Steinacker J, Schabana R, Hamich S, et al. Arterial blood gases during diving in elite apnea divers. Int J Sports Med. 2003; **24**(2): 104-7.

**Appendix S1.** National Institutes of Health Stroke Scale (NIHSS)

**Appendix S2.** Activities of daily living (ADL) questioner

**Appendix S3.** EQ-5D questioner
