# Supplementary material for: Hyperbaric Oxygen Induces Late Neuroplasticity in Post Stroke Patients - Randomized, Prospective Trial
Source: PLoS One. 2013 Jan 15;8(1):e53716. doi: 10.1371/journal.pone.0053716 (PMC3546039; doi:10.1371/journal.pone.0053716)
Supplement: Text S1 — Statistical, Randomization and Placebo Considerations. (DOCX) [file pone.0053716.s004.docx]

S2: Statistical, Randomization and Placebo Considerations

**1. Statistical Considerations**

**1.1 The analysis sets**

**Safety analysis set:** This set will consist of all subjects for whom the study treatment was initiated.

**Primary efficacy:** The co-primary endpoints measured in this study are: improvement in NIHSS tests score and improvement in ADL score following HBOT treatment for post stroke patients suffering chronic neurological deficiencies.

The primary efficacy analysis evaluation will include all For 80% power to detect a difference of 25% between study and control group on the NIHSS test score, with 1:1 random allocation using a one sided *t* test with α= 0.05, a sample size of 31 patients (for each group) would be required.

NIHSS test, ADL and quality of life scores of treated and cross groups were compared at baseline and following HBOT treatment (for treatment group). Furthermore, the scores of the cross group at baseline were compared to NIHSS test scores obtained following a no treatment period, and following HBOT treatment using One Way ANOVA. Categorical data is expressed in numbers and percentages and compared by chi-square test. P values<0.05 were considered significant.

All randomly allocated patients were included in the safety analysis.

**1.2 Sample size considerations**

Presentation of sample size is based on achieving 80% power overall to demonstrate that improvement rate in *NIHSS test score* is at least 0.25 and, separately, that improvement rate in *ADL test score* is at least 0.25. An improvement rate of at least 25% in the NIHSS test and ADL score in the treatment group (comparing to baseline, prior to HBOT treatment) would be an appropriate clinical target for HBOT treatment success and of sufficient interest likely to encourage further investigation of HBOT treatment for post stroke patients suffering chronic NIHSS deficiencies.

Sample size was based on the assumption that exposure to the NIHSS tests and ADL evaluation (at baseline) without any additional training might induce up to 4% score improvement in the second NIHSS test (following treatment). Assuming a true success rate of 25% a sample of N = 31 patients (for each group) will provide 80% power to show that HBOT treatment induces at least 25% improvement on *NIHSS test score* and separately, on *ADL test score*. This is based on a power analysis with 1:1 random allocation using the normal approximation for the binomial, with one-sided Alpha=0.05.

**2. Randomization and Placebo Considerations**

**2.1 Randomization**

Since the diversity of the patients included in the study was expected to be high, no stratification criteria were used. Patients were randomized in 1:1 fashion to either the treatment group or the controlled-cross group.

**2.2 Placebo constraints**

There is an inherent constraint to carrying out a direct placebo in HBOT study. The patients in the chamber detect if the pressure is increased or not. In principle, it is possible to carry out a partial placebo: to place the patients in the chamber and increase the pressure without increasing the oxygen concentration in the air. However, the amount of dissolved oxygen in the blood will increase as a result of the pressure increase (according to Henry’s law^1^). Hence, the only option is to perform a direct “negative placebo” by increasing the pressure while lowering the oxygen concentration in the air, which holds significant safety hazards and brings up serious ethical questions.

In order to partially compensate for the absence of placebo, the patients in the cross group went through 2 months with no treatment at all followed by a second evaluation and then crossed to HBOT. The cross for treatment enables intra-group efficacy evaluation in both treatment and control periods in the same group and compare the results to the patients in the immediately-treated group.

**2.3 Blinding constraints**

Since the patients knew they were not receiving HBOT during the control period, they were not blinded with regard to the treatment arm. Accordingly, the ADL and quality of life evaluations were not blinded. Moreover, in the current clinical setting, it was not possible to assure that the NIHSS evaluation could be done completely blinded by the physician. The complete blindness evaluation could be assured in the brain SPECT evaluation done by the radiologist. The correlation between metabolic blinded evaluation of the brain (SPECT) and the neurological un-blinded evaluations give further support and strength to the clinical findings.

**References**

1. Muth CM, Radermacher P, Pittner A, Steinacker J, Schabana R, Hamich S, et al. Arterial blood gases during diving in elite apnea divers. Int J Sports Med. 2003; **24**(2): 104-7.
