# Supplementary material for: Hyperbaric Oxygen Induces Late Neuroplasticity in Post Stroke Patients - Randomized, Prospective Trial
Source: PLoS One. 2013 Jan 15;8(1):e53716. doi: 10.1371/journal.pone.0053716 (PMC3546039; doi:10.1371/journal.pone.0053716)
Supplement: Text S2 — Scatter plot analysis of the clinical scores. (DOCX) [file pone.0053716.s005.docx]

S5: Scatter plot analysis of the clinical scores

The analysis is aim at better quantifications of the changes in the clinical scores (NIHSS, ADL, EQ-5D and EQ VAS) while taking into consideration the high patient-to-patient variability in each of these clinical scores and to compare between the changes in the different scores. The idea is to inspect for each patient the scaled relative differences of each of the clinical scores (in comparison to all the patients in the group), for each time stage (control or treatment).

**1. Construction of the scatter plots**

First we calculate for each specific patient (j) the relative difference in the clinical score, RD_j_, defined as

RD_j_ ≡ (SF_j_ – SI_j_)/[1/2(SF_j_ + SI_j_)]

Where (SF_j_) is the value of a clinical score at the end of the time stage and (SI_j_) is the score value at the beginning of the time stage.

While this analysis enables quantitative inspection of the changes in the clinical scores, the results can be further improved when the relative differences are scaled by the standard deviations (STD) of the score. So next we calculate for each patient (j), the corresponding scaled relative difference SRD_j_ defined as

SRD_j_ ≡ (SF_j_ – <SF_j_>)/STD(SF_j_) - (SI_j_ – <SI_j_>)/STD(SI_j_) + <SF_j_ – SI_j_>)/STD(SF_j_ - SI_j_)

We note that the averaged difference (<SF_j_–SI_j_>) is not divided by STD(SF_j_-SI_j_).

**2. Results**

Scatter plot analysis of the changes in the quality of life evaluations in the different study group are represented in figure 1. The difference in the results between the treated periods (of the treated and cross group) to the cross group during the control period is clearly visualized in particular when inspecting the scaled relative differences SRD_j_. Despite the higher patient-to-patient variability in the quality of life results in comparison to the results of the neurological evaluations (shown in Figure 3 in the main text), that are due to the more subjective nature of the former, the different between the treated periods to the cross group during the control period is clearly visualized. It can also be visualized that with respected to improvement there was no statistical different between the treated group and the cross group after the HBOT. Meaning the HBOT had similar beneficial effect in the treated and the cross group after the cross to the HBOT.

**
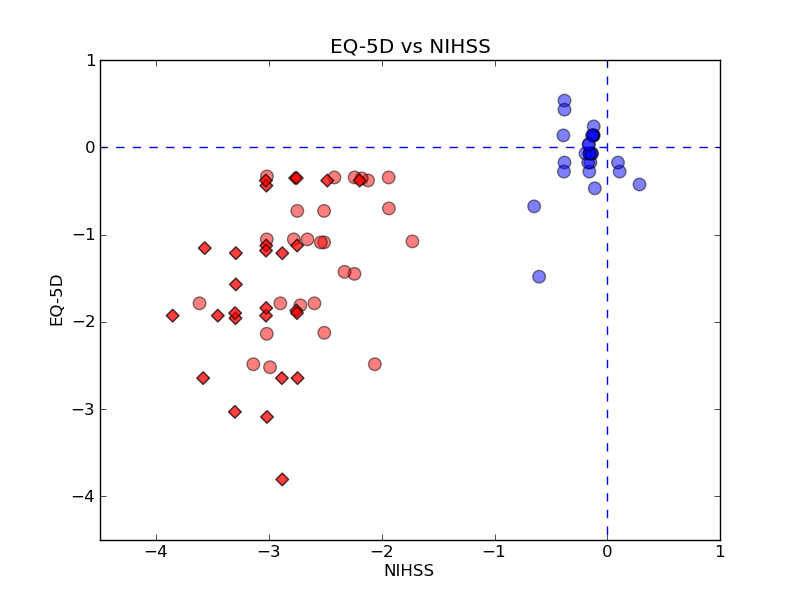

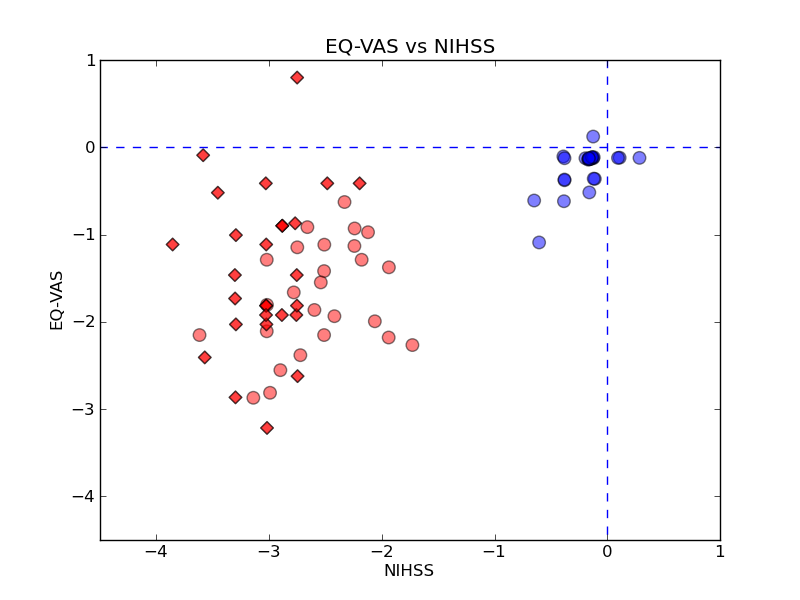
**

**-A- -B-**

**Figure S1:** Scatter plot analysis of the changes in the quality of life scores (EQ-5D and EQ-SAV) in comparison to the neurological score (NIHSS). The results of the scaled relative differences SRD for the different study groups at the different periods of the study are shown using the same symbols and color code at in Figure 3 (for ADL as function of NIHSS) in the main text: HBOT-treated group (red diamonds), HBOT-treated cross group (red circles) and control (non-treatment) period of the HBOT-treated cross group (blue circles).
